# Chemical modification of MTA and CEM cement to decrease setting time and improve bioactivity properties by adding alkaline salts

**DOI:** 10.34172/joddd.2020.001

**Published:** 2020

**Authors:** Faeze Jamali Zavare, Hanieh Nojehdehian, Maryam Moezizadeh, Mehdi Daneshpooy

**Affiliations:** ^1^Department of Operative Dentistry, School of Dentistry, Shahid Beheshti University of Medical Sciences, Tehran, Iran; ^2^Irainin Center for Endodontic Research, Research Institute of Dental Sciences, Shahid Beheshti University of Medical Sciences, Tehran, Iran; ^3^Department of Dental Biomaterials, School of Dentistry, Shahid Beheshti University of Medical Sciences, Tehran, Iran; ^4^Department of Operative Dentistry, Dental School, Shahid Beheshti University of Medical Sciences, Tehran, Iran; ^5^Department of Restorative Dentistry, Faculty of Dentistry, Tabriz University of Medical Sciences, Tabriz, Iran

**Keywords:** Calcium-enriched Mixture cement, calcium ion release, hydroxyapatite, MTA, setting time

## Abstract

***Background.*** Mineral trioxide aggregate (MTA) and Calcium-enriched Mixture (CEM) cement are used for pulp capping since they induce the formation of a dentinal bridge. Long setting time is a shortcoming of these types of cement. This study aimed to assess the effect of the incorporation of some alkaline salts to MTA and CEM cement on their setting time, ion release profile, pH, and surface morphology.

***Methods.*** In this in vitro experimental study, 5% calcium chloride (CaCl_2_), calcium oxide (CaO), sodium fluoride (NaF), and calcium nitrate [Ca(NO_3_)_2_] were separately added to MTA and CEM cement. The primary and final setting times of the cements were measured using a Gillmore needle apparatus. The samples were immersed in simulated body fluid (SBF) for one, seven, and 14 days and subjected to x-ray diffraction (XRD) and scanning electron microscopy (SEM) for phase identification and surface morphology assessment. The change in the pH of solutions was studied, and the calcium ion release profile was determined using inductively coupled plasma atomic emission spectroscopy (ICP-AES). The data were analyzed with ANOVA, followed by post hoc tests.

***Results.*** CaCl_2_ and CaO decreased the setting time of MTA, and Ca(NO_3_)_2_ decreased the setting time of CEM cement. The incorporation of the salts increased the pH and calcium ion release from both cements, and hydroxyapatite deposits were noted to cover the surface of the samples (observed by SEM and confirmed by EDXA).

***Conclusion.*** The incorporation of CaCl_2_ and CaO into MTA and Ca(NO_3_)_2_ into CEM cement decreased their setting time and increased their pH and calcium ion release.

## Introduction

The first formulation of mineral trioxide aggregate (MTA), introduced under the brand name of ProRoot MTA, contained a mixture of Portland cement and bismuth oxide with a 4:1 ratio.^1^ This cement had optimal properties for endodontic treatment of teeth, such as polymerization in a wet environment, favorable marginal fit,^2^ optimal dimensional stability, antimicrobial activity, high alkalinity, biocompatibility,^3^ and bioactivity.^4^ Evidence shows that ProRoot MTA can react with fluids containing phosphate to form a mineral deposit on the surface.^5^ A superficial layer forms between the tooth structure and cement, and the minerals deposit into the dentinal tubules, referred to as biomineralization.^6^ Biocompatibility and bioactivity of ProRoot MTA cement have been confirmed in previous studies.^5-8^ It can stimulate the proliferation of pulp cells^9^ and induce their differentiation,^10^ with no adverse effects on cell survival.^10,11^ However, it has two main shortcomings, namely poor handling and long setting time (165 minutes).^12^ Several calcium silicate-based cements, including MTA Angelus, were marketed to overcome these shortcomings. MTA Angelus can also stimulate the biomineralization process; it is also biocompatible and bioactive.^13-16^ According to the manufacturer, the primary setting of this cement occurs within 15 minutes. This fast setting occurs due to the absence of sulfur and sudden hydration of tricalcium aluminate. Although its primary setting occurs faster than ProRoot MTA, it takes 230 minutes for this cement to reach its final physical properties due to the delayed hydration process of calcium silicate particles.^17^

Calcium-enriched Mixture (CEM) cement, introduced in 2006 as a root filling material,^18^ also has optimal physical properties, such as adequate flow, film thickness, and primary setting time.^19^ It induces hard tissue formation.^20^ It sets in wet environments and has sealing properties comparable to those of MTA with superior antimicrobial properties.^21,22^ Although its setting time is shorter than that of MTA (about one hour),^23^ it would be ideal for enhancing its setting reaction further.

Since the fast setting of these cement types is vital in endodontic surgery and pulp capping procedures, it is imperative to find a formulation of cements with short setting time and optimal biocompatibility. It has been suggested that some accelerators can decrease the setting time of cements. Calcium chloride (CaCl_2_) was suggested as the most effective accelerator for Portland cement.^24^ However, chlorides produced by the reaction of this cement increase the corrosion of the cement.^24^ Calcium oxide (CaO) has also been suggested to enhance the setting reaction of cements. However, it has large particles that might remain unreacted during the hydration phase. It also increases the solubility of cements and their alkalinity.^25^ Calcium nitrate [Ca(NO_3_)_2_] is another accelerator, which has high solubility and decreases the porosity of cements. Its efficacy for the enhancement of setting reactions is similar to that of CaCl_2_, but it is more expensive than that. Its toxicity should also be taken into account. Its efficacy seems to depend on the composition of the cement. It has been reported to decrease the setting time by 20% when added to a cement containing tricalcium silicate with no effect on the long-term strength of the cement.^22^

Considering the significance of a short setting time of these cements in the clinical setting, this study aimed to assess the effect of the incorporation of different alkaline salts into MTA and CEM cement on their physical properties, such as setting time, pH, calcium ion release, and apatite formation.

## Methods

This study had an in vitro experimental design. Table 1 shows the groups and materials used in this study. The samples were divided into 10 groups, as follows (Table 1):

**Table 1:** Group characteristics in this study

**Group**	**Dental Cement**	**Additive**
**1**	MTA (Angelus, Brazil)	-
**2**	MTA (Angelus)	CaCl_2_ (Merck)
**3**	MTA (Angelus)	NaF (Sigma-Aldrich)
**4**	MTA (Angelus)	CaO (Merck)
**5**	MTA (Angelus)	Ca(NO_3_)_2_ (Merck)
**6**	CEM Cement (BioniqueDent , Iran)	-
**7**	CEM Cement	CaCl_2_
**8**	CEM Cement	NaF
**9**	CEM Cement	CaO
**10**	CEM Cement	Ca(NO_3_)_2_

### Preparation of the cements

One scoop of the powder and one drop of the liquid were weighed according to the manufacturer’s instructions for MTA cement preparation; the powder-to-liquid ratio was 3:1. The powder-to-liquid ratio for the CEM cement was 2:1, according to the manufacturer’s instructions.

All the salts were added to the cement powder except for Ca(NO_3_)_2_, which was added to the liquid due to its large crystals.

Prior to mixing, the spatula and the glass slab were placed at 23±1°C for one hour; then, the powder and liquid were mixed on a clean glass slab with a spatula in 30 seconds to obtain a homogeneous paste with a thick creamy consistency. After mixing, the samples were placed in molds.

### Assessment of calcium ion release

In each of the MTA and CEM cement groups, the samples were immersed in simulated body fluid (SBF) for one, seven, and 14 days and then incubated at 37°C under 95% humidity.

After the removal of the samples, the SBF was filtered and analyzed using inductively coupled plasma atomic emission spectroscopy (ICP-AES) (ICP-AES3410 ARL; Switzerland) to determine the amount of calcium ions released from each sample into the solution. The Ca release concentration were the average values of the three samples (mean ± SD).

### Assessment of the setting time using the Gillmore needle apparatus

The samples of MTA and CEM cement (n=3 in each group) were prepared, as explained in the section on the preparation of the cement. A powder-to-liquid ratio of 3:1 was considered for MTA, with a powder-to-liquid ratio of 2:1 for the CEM cement, according to the manufacturer’s instructions.

The duration of the reaction was measured from the beginning of mixing the powder and liquid using a chronometer. After complete mixing of the cement, the paste was placed in cylindrical plastic molds with a diameter of 1 cm and a height of 3 mm. After 30–60 seconds since the initiation of mixing, the samples were placed in the Gillmore needle apparatus, with the needle being inserted vertically into the surface of the material. The primary setting time was defined as the time from the termination of mixing until the tip of the Gilmore needle no longer caused an indentation on the surface, according to ISO 6876 2001. This device has a cylindrical needle with a 2-mm diameter and 5-cm length that applies a load of 100±0.5 g to the cement surface. The testing temperature was 37±1°C under a relative humidity of 95%. A cylindrical needle, measuring 2 mm in diameter and 5 cm length, applied a load of 450±0.5 g to the surface to measure the final setting time. The setting time mean values were reported (mean ± SD).

### X-ray Diffraction (XRD)

After mixing the powder and liquid, the mixture was transferred into plastic molds, and a moist piece of gauze was placed over it. The samples were then incubated at 37°C under 95% relative humidity for 24 hours for complete setting. Then, they were soaked in 10 mL of SBF and incubated again. In each group, three samples were immersed for one, seven, and 14 days in SBF. After removal, they were rinsed with deionized water, dried in an incubator and mounted on the x-ray diffraction device (Equinox 3000, Inel, France) with a 5–118° scanning angle, 1.54187 Å wavelength, 40 kVp, and 30 mA in the range of 20<2θ<60 for phase identification. The patterns were identified using JCPDS reference carts and X’Pert High Score software.

### Scanning Electron Microscopy (SEM)

The samples were fabricated for SEM analysis, as explained for XRD. The surfaces of the samples were inspected under SEM after the setting test and immersion for one, seven, and 14 days in SBF. For this purpose, the samples were gold-coated and evaluated under a scanning electron microscope (EM3200; KYKY, China) at 25 kVp. The data were subjected to energy-dispersive x-ray spectroscopy (EDS) using a Vega electron microscope (Tescan).

### pH variations

The pH of the samples immersed in SBF was measured at 1-, 7-, and 14-day intervals using a pH meter (Jenway 3310, UK). The pH variation tests were repeated three times for further accuracy.

### Statistical analysis

The data were analyzed with ANOVA, followed by post hoc tests at a significance level of P<0.05.

## Results

### Release of calcium ions

**MTA cement:** On day one, the release of calcium ions in (MTA + CaCl_2_) and [MTA + Ca (NO_3_)_2_] groups was not significantly different from that in the MTA group (P>0.05). The group with MTA + 5% NaF showed higher calcium release than the MTA group (P=0.000; Table 2). On day seven, the calcium release in all the groups was significantly higher than that in the MTA group (P=0.000). On day 14, calcium release in the group with MTA + CaCl_2_ was not significantly different from that in the MTA group (P>0.05). The calcium release between the three groups of CaCl_2_, NaF, and CaO was significantly different. MTA cements containing CaCl_2_ and CaO exhibited higher, and NaF exhibited less calcium release compared to the control group.

**Table 2:** The release of calcium ion from MTA and CEM cement groups

	**Group**	**ICP, day 1**	**ICP, day 7**	**ICP, day 14**
**1**	**MTA**	281±8	318±15	761±34
**2**	**MTA + 5% CaCl**_2_	268±16	607±8	810±15
**3**	**MTA + 5% NaF**	146±8	456±7	485±11
**4**	**MTA + 5% CaO**	311±5	495±2	830±9
**5**	**MTA + 5% Ca( NO**_3_ **)**_2_	272±6	390±6	622±6
**6**	**CEM**	215±6	482±6	508±9
**7**	**CEM + 5% CaCl**_2_	257±9	622±8	851±16
**8**	**CEM + 5% NaF**	170±7	623±19	724±13
**9**	**CEM + 5% CaO**	230±7	544±22	698±21
**10**	**CEM + 5% Ca( NO**_3_ **)**_2_	242±5	504±16	705±12

Adding salts [CaCl_2_, CaO, Ca(NO_3_)_2_] led to a significant increase in the release of calcium during two weeks of immersion in SBF (P<0.05).

**CEM cement:** On day one, incorporation of CaCl_2_ and Ca(NO_3_)_2_ resulted in significantly higher calcium release compared to the CEM group (P<0.05). The cements that contained NaF exhibited less calcium release. After one week, groups 7, 8 and 9 (CEM + CaCl_2_, CEM + NaF and CEM + CaO) showed significantly higher calcium release compared to the CEM group (P<0.05), and group 10 [CEM + Ca(NO_3_)_2_] exhibited no significant difference from the CEM group (P>0.05; Table 3). On day 14, all the groups showed higher calcium release than the CEM group.

**Table 3:** The primary and final setting times of MTA and CEM cements

	**Group**	**Primary setting time (minutes)**	**Final setting time (minutes)**
**1**	**MTA**	11.16±0.66	20.1±0.7
**2**	**MTA + 5% CaCl**_2_	12.81±1	14.93±1
**3**	**MTA + 5% NaF**	25.06±1	37.53±0.5
**4**	**MTA + 5% CaO**	11.55±1	14.08±0.7
**5**	**MTA + 5% Ca( NO**_3_ **)**_2_	14.33±1	27.13±0.6
**6**	**CEM**	27±0.38	36.38±2.68
**7**	**CEM + 5% CaCl**_2_	20.3±0.28	33.23±0.98
**8**	**CEM + 5% NaF**	23.11±0.56	51.03±0.94
**9**	**CEM + 5% CaO**	33.62±0.58	40.21±0.94
**10**	**CEM + 5% Ca( NO**_3_ **)**_2_	11.05±0.98	32.28±0.93

In all the groups, calcium release increased from day one to day 14, and the differences between days one and seven, one and 14, and seven and 14 were statistically significant.

### Setting time

**MTA cement :** The primary setting time in groups 3 (MTA + 5% NaF) and 5 [MTA + 5% Ca(NO_3_)_2_] significantly increased (P<0.05). In groups 2 (MTA + 5% CaCl_2_) and 4 (MTA + 5% CaO), the primary setting time increased, but it was not significant (P>0.05; Table 3).

The final setting time of the four experimental MTA groups was significantly different from that of the control group (MTA) (P<0.05). A reduction in setting time was noted in groups 2 (MTA + CaCl_2_) and 4 (MTA + CaO) (P>0.05), while an increase in setting time was noted in groups 3 and 5 (MTA + NaF) and [MTA + Ca(NO_3_)_2_] (P>0.05).

**CEM cement:** The initial setting time in groups 7 to 10 was significantly different from the CEM group (P<0.05). Group 9 (CEM + CaO) showed a longer setting time than the CEM group (P>0.05), while the setting time decreased in other groups (P>0.05). The final setting times of groups 7 and 10 were not significantly different from that of the CEM group (P>0.05). In the CEM + NaF group, the final setting time was longer than that of the CEM group, while the final setting time decreased in group 10 [CEM + Ca(NO_3_)_2_] (P>0.05).

### SEM and XRD results

**Figure 1:**
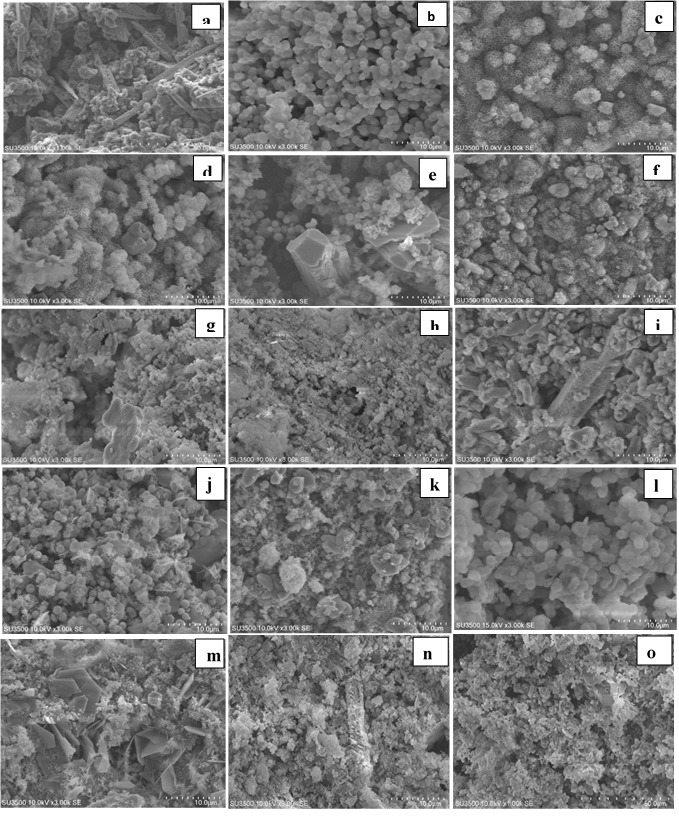
Scanning electron micrographs of MTA cements after immersion in SBF for 1, 7th and 14th days (MTA: a,b,c);( MTA + CaCl2:d, e,f) ; (MTA+NaF:g,h,i) ;(MTA+ CaO:k,j,l) ;(MTA+Ca(NO3)2: m,n,o)

**MTA cement:** Figures 1 and 3 show the results of SEM and XRD analyses of each group. As shown, hydration reaction products formed completely in all the groups. Since calcium silicate hydrogel (CSH) is amorphous, the peaks indicative of this product were almost unidentifiable, and CSH gel had weak peaks at 32.28°, 39.89°, and 29.76°; the intensity of these peaks was very low. The peaks belonging to calcium hydroxide were noted at 33.85°.

**Figure 2:**
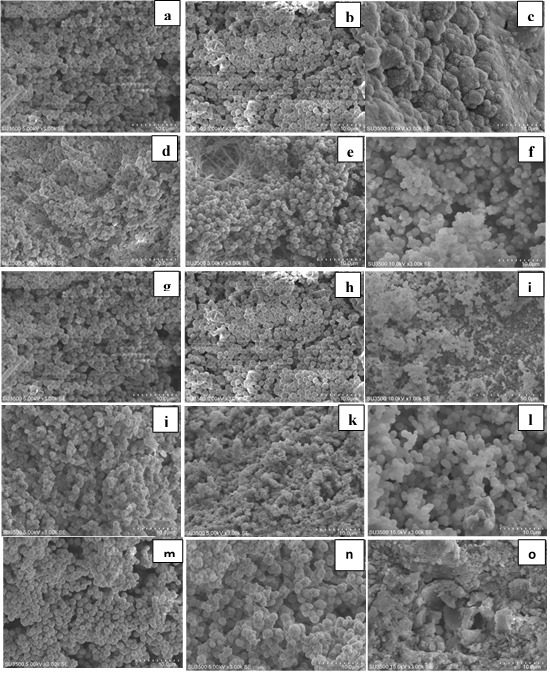
Scanning electron microscopy of CEM cements after immersion in SBF for 1,7th ,14th days (CEM: a,b,c ) ;(CEM + CaCl2:d,e,f); (CEM+NaF:g,h,i); (CEM+ CaO: j,k,l); (CEM+Ca(NO3)2:m,n,o)

**Figure 3:**
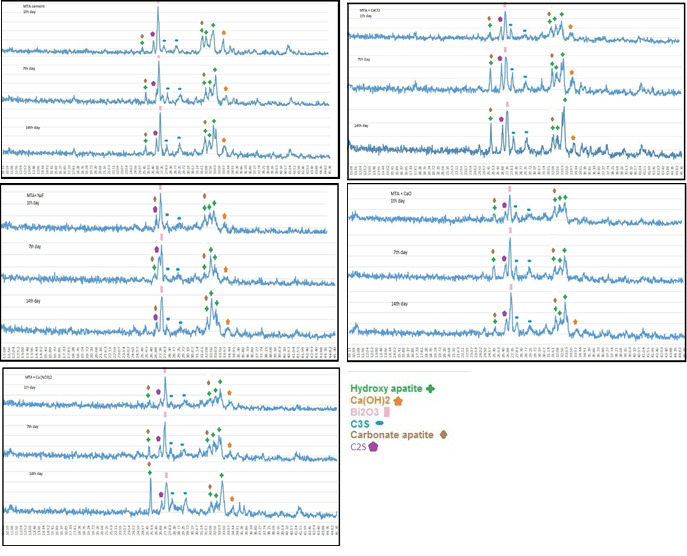
XRD patterns of cements after MTA cements after immersion in SBF for 1, 7 and 14 days.

**Figure 4:**
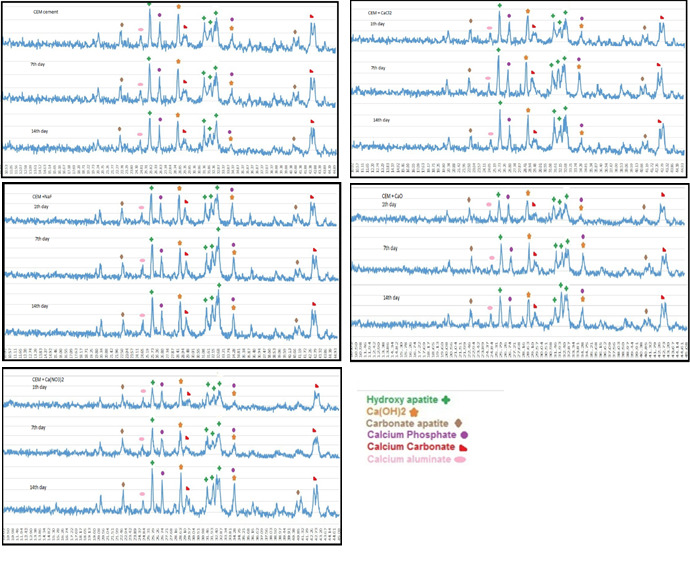
XRD patterns of cements after CEM cements after immersion in SBF for 1, 7 and 14 days.

In all the five groups, on day one, SEM micrographs showed the CSH matrix along with porosities and scattered calcium hydroxide crystals. Following hydration reactions of calcium silicate cement, some tricalcium silicate remained unreacted with peaks at 29.7° and 27.29°. Hydroxyapatite peaks were noted at 31.73°, 32.86°, 32.18°, and 25.87°. This indicates that hydroxyapatite crystals formed on the cement surface.

SEM micrographs showed that the cement surface was covered with a layer of spherical hydroxyapatite crystals. This layer was seen since the first day of immersion in SBF, and its thickness increased over time (days seven and 14). On day 14, this layer had the greatest thickness. The results of XRD showed that the amount of hydroxyapatite formed on the cement surface increased over time, and the intensity of peaks showed an ascending trend. As shown on SEM micrographs, MTA cement had an irregular surface with unreacted particles on day one. Following immersion of cement in SBF for seven days, hydroxyapatite crystals deposited on the cement surface and covered its porosities. Carbonate apatite crystals were also noted with peaks at 25.9° and 31.73°. According to the results of XRD, the intensity of these peaks was higher on day 14 compared to days seven and one. In all the groups, the amount of hydroxyapatite seen on SEM micrographs on day 14 was higher than that on day seven, and the latter was higher than that on day one. In addition, in all the four groups with salts, higher amounts of hydroxyapatite were noted on day one compared to the control group. In all the groups, apatite crystals covered the cement surface completely on days seven and 14. Also, according to the results of XRD, calcium hydroxide peak on day seven was higher than that on day one but decreased on day 14 (probably due to the dissolution of calcium hydroxide and conversion to hydroxyapatite).

**CEM cement:** Figures 2 and 4 show the results of SEM and XRD analyses of CEM groups. The peaks for calcium hydroxide were noted at 28.55° and 34.8°. The peaks for hydroxyapatite were noted at 31.73°, 32.86°, and 25.87°. Other compounds included calcium carbonate with peaks at 29.2° and 42.7°, calcium phosphate with peaks at 26.51° and 34.26° and calcium aluminate with a peak at 24.71°. In these groups, hydroxyapatite crystals were formed on the cement surface. As seen in Figure 2, the cement surface was covered with hydroxyapatite crystals. This layer was observed since the first day of immersion in SBF. Over time, its thickness increased (days seven and 14). On day 14, this layer had the highest thickness. The results of XRD confirmed that the amount of hydroxyapatite formed on the cement surface increased over time, and the intensity of these peaks increased, as well. Also, carbonate apatite peaks were noted at 22.8° and 40.7°.

On day one, all the groups containing salt showed higher apatite than the control group on their surface. However, in the control group, there was a small amount of apatite on day one. On days seven and 14, the surface of all cements was covered with apatite crystals.

### pH variations

The pH of MTA increased over time. On day one after immersion in SBF, the pH of all the groups was significantly higher than that of the control group except for group 4 (P<0.05). On day seven, only group 2 exhibited significantly higher pH than the control group (P<0.05), and the remaining groups were not significantly different from the control group (P>0.05). On day 14, the pH of all the groups was significantly higher than that of the control group (P<0.05).

In the control group and groups 2 and 4, the pH on days one and seven (P>0.05), one and 14 (P<0.05) and seven and 14 (P<0.05) was significantly different, but the difference between days seven and 14 (P>0.05) was not significant. The highest pH in group 2 (MTA + 5% CaCl_2_) was noted on days one, seven, and 14. Table 4 shows changes in pH in the MTA groups.

**Table 4:** The pH variation in MTA and CEM cements after immersion in SBF for 1, 7 and 14 days

	**Group**	**pH on day 1**	**pH on day 7**	**pH on day 14**
**1**	**MTA**	7.98±0.01	8.55±0.02	9.15±0.15
**2**	**MTA + 5% CaCl**_2_	8.04±0.02	8.71±0.09	9.32±0.2
**3**	**MTA + 5% NaF**	8.32±0.02	9.00±0.01	9.36±0.01
**4**	**MTA + 5% CaO**	8.04±0.005	8.65±0.1	9.3±0.2
**5**	**MTA + 5% Ca( NO**_3_ **)**_2_	7.97±0.02	8.46±0.11	9.21±0.1
**6**	**CEM**	7.66±0.01	8.03±0.02	8.3±0.01
**7**	**CEM + 5% CaCl**_2_	8.26±0.01	8.9±0.04	8.95±0.06
**8**	**CEM + 5% NaF**	7.81±0.009	8.85±0.04	9.29±0.32
**9**	**CEM + 5% CaO**	8.43±0.01	8.88±0.08	9.2±0.05
**10**	**CEM + 5% Ca( NO**_3_ **)**_2_	8.28±0.01	9.1±0.1	9.31±0.09

The pH of all the CEM groups on days one, seven, and 14 was significantly higher than that of the control group (P<0.05). The difference in pH between days one, seven, and 14 was significant within each of the five groups (P<0.05). The pH increased over time.

In the control group and groups 8 (CEM + NaF) and 9 (CEM + CaO), there were significant differences between days one and 14 (P<0.05), one and seven (P<0.05), and seven and 14 (P<0.05). In groups 6 (CEM) and 7 (CEM + CaCl_2_), days one and seven (P<0.05) and days one and 14 (P<0.05) were significantly different in this respect, with no difference between days seven and 14 (P>0.05). The highest pH was noted in group 10 [CEM + Ca(NO_3_)_2_] on days seven and 14 (Table 4).

## Discussion

This study aimed to assess the effect of adding some alkaline salts to MTA and CEM cement on their setting time, calcium ion release profile, pH, and dentinal bridge formation. The results showed that the incorporation of 5% CaCl_2_ into MTA decreased its final setting time, with no significant effect on its initial setting time. Following the addition of this salt, the cement required less water for setting, and thus, fewer bubbles formed in its structure. Therefore, the solubility of the cement decreased, and its mechanical properties improved. CaCl_2_ entered the cement surface porosities and not only affected the tricalcium silicate but also affected the hydration reaction of other components, such as CaO, resulting in the formation of greater amounts of Ca(OH)_2_.^1^

Calcium nitrate (at 5–20°C) can enhance the setting reactions of the cement, depending on its composition. According to Ouki and Hills, small amounts of nitrate can decrease the porosities in cement and decrease the hydration by 50%.^26^ These findings were confirmed in the present study (an increase in both primary and final setting of the cement). According to Chik et al, the presence of 8.33% aluminate only slightly decreased the setting time while 2.82% aluminate decreased the primary and final setting time by 22%.^27^ Camilleri et al^28^ reported that incorporation of calcium nitrate decreased the primary setting of Portland cement, with no effect on the primary setting of white MTA.

In the present study, the incorporation of 5% CaO into MTA cement did not affect its primary setting time but decreased its final setting time. According to Camilleri et al,^28^ the main problem of MTA Angelus is a defect in the primary mixing of ingredients and the fabrication of its powder. If the sintering temperature is not high enough, the process of the powder (clinker) fabrication will not proceed correctly, and the size of C3S and C2S particles will remain small. In addition, CaO and SiO_2_ remain inactive due to inadequate amounts of C3S and C2S. The incorporation of CaO probably increases the chances of the reaction of these particles, decreasing the final setting of the cement. According to Camilleri et al,^28^ CaO has large particles and may not well react during hydration.The lack of change in the primary setting despite a reduction in the final setting might be explained by a delay in the onset of CaO particles’ reaction.

The incorporation of 5% NaF into MTA cement increased the primary and final setting of MTA. This delay in the setting of cement is due to the fluorine ions that react with calcium ions to form CaF_2_, which is insoluble, is deposited on the cement surface, and increases its water sorption. As a result, the solubility/hydration and ion movement increase. In addition, the reaction of silica ions with fluorine results in the formation of calcium silica fluorine (containing SiF_6_ ions) and delays the setting reaction of the cement.

In a study by Gandolfi et al,^29^ the incorporation of NaF into MTA increased the primary setting and decreased the final setting time. However, they used ProRoot MTA, which contains sulfur, and its setting mechanism is slightly different from that of MTA Angelus.

In the present study, XRD and SEM analyses showed that after immersion of the cements in SBF, an apatite biolayer formed on the surface of the cements. When the concentration of phosphate is low in the solution, the rate of deposition of apatite decreases, and the time required for the formation of the superficial layer increases. Gandolfi et al,^29^ evaluated the behavior of calcium silicate cement following immersion in DPBS using eSEM and showed that the cement surface was smooth at first and covered with a layer of water. The porosities were filled with a silica-rich gel (Si-OH gel). After 5–24 hours, a thin layer of spherical crystals accumulated on the surface. These apatite particles were composed of calcium and phosphate. After 14–28 days, several layers of spherical hydroxyapatite crystals formed on the surface, consistent with the findings in the present study. Oliviera et al^30^ showed that Si-OH functional groups provided suitable areas for the budding of hydroxyapatite crystals. It has also been reported that silica particles enhance further deposition of apatite particles on the cement surface.^19^ It appears that immersion in SBF changes the morphology and texture of the surface, the size of deposited crystals, and the depth of porosities.^31^ It seems that Si-OH groups on the surface of freshly set cements serve as a catalyzer for the deposition of calcium phosphate films. Calcium required for this process is obtained from the dissolution of calcium hydroxide in the solution while phosphate ions are provided by the solution itself.^31^

For the assessment of calcium ion release, ICP-AES was used in the present study, which has a high speed and optimal accuracy (ppm level). The results showed that calcium ion release in all the MTA samples significantly increased from day one to 14, and the incorporation of salts had no adverse effect on this process. CaCl_2_ and CaO showed higher calcium release compared to others, which is probably due to the presence of calcium ion in their composition. Bortoluzzi et al^26^ reported that the incorporation of CaCl_2_ into Portland cement increased pH and the release of calcium and enhanced its bioactivity. NaF salt can also form hydroxyfluorapatite. The results of the present study showed that the pH of samples also increased from day one to day 14. The absence of any increase in pH from day seven to 14 was probably due to the fact that calcium-based compounds tend to decrease the ionization of calcium hydroxide and thus, calcium and OH^–^ ions (that increase the pH) are produced in lower amounts.^32^ The cement has antibacterial activity in this range of pH, enabling the deposition of hydroxyapatite and the formation of a dentinal bridge. XRD results confirmed that calcium hydroxide was produced by the hydration reaction of the cement, and after dissolution in SBF, it reacted with phosphate ions to produce hydroxyapatite. Filho et al^33^ showed that the incorporation of 20% CaO, along with niobium and zirconium oxide, into Portland cement, increased its pH.

MTA Angelus contains C3S, C2S, C3A, and bismuth oxide. SiO_2_ provides an optimal basis for the nucleation and deposition of apatite crystals. First, C3S particles react with water to form a calcium silicate hydrate (CSH) matrix and calcium hydroxide, which was confirmed by XRD in all the samples in the present study. These reactions are responsible for the primary mechanical properties of the cement. Some tricalcium silicate remains unreacted with peaks at 32.12°, 32.46°, and 27.29°. The hydration of C2S continues for a couple of days, contributing to the final mechanical properties of the cement. SEM micrographs showed that the MTA cement surface was irregular on day one and had unreacted particles. By longer immersion in SBF, a thin white layer of the deposit formed on the surface, which was confirmed to be hydroxyapatite chemically (as confirmed by XRD) and structurally (as confirmed by SEM). The evaluation of apatite peaks on day one showed that the formation of apatite was higher in salt groups compared to the control group. SEM micrographs also confirmed the presence of higher amounts of apatite on the surface of samples containing salts. After seven days, hydroxyapatite particles covered the cement surface and porosities. Some carbonate apatite crystals were also noted. XRD showed a higher intensity of these peaks after 14 days, which was confirmed by SEM observations. SEM micrographs revealed that calcium phosphate apatite deposits filled the porosities and covered the surface, creating a rather irregular form. After seven and 14 days, the surface of the samples was covered with a thick layer of spherical apatite granules. In the clinical setting, this layer can enhance the differentiation of osteoblasts and their adhesion. ^33^

Regarding CEM cement, the results of the present study showed that the incorporation of 5% CaCl_2_ decreased the primary setting time of CEM, with no effect on its final setting time. In a similar study, Abbaszadegan et al^34^ showed that the incorporation of 10% CaCl_2_ decreased the primary setting time of CEM cement, with no effect on its final setting time, which was consistent with the results of the present study. The incorporation of CaCl_2_ probably affects CaO and increases the release of calcium ions and the formation of calcium hydroxide and enhances the setting reaction of the cement. In addition, it might increase the temperature of the reaction and expedite the process as such. Different results obtained by the incorporation of salts into CEM and MTA are probably due to the differences in the compositions of these two cements since MTA is a calcium silicate, and CEM is a calcium phosphate cement. Moreover, these two cements are different in terms of the quantity of compounds (such as tricalcium silicate) in their compositions. The incorporation of Ca(NO_3_)_2_ into CEM cement decreased both its primary and final setting times. An increase in calcium ions following the incorporation of this salt probably increases the formation of calcium hydroxide and enhances the setting of the cement. In addition, an increase in temperature might have enhanced the setting reaction. The incorporation of 5% CaO also increased the primary setting of CEM, with no effect on its final setting. The incorporation of 5% NaF into CEM decreased its primary setting and increased its final setting.

A likely scenario is that the incorporation of this salt probably replaced OH^–^ ions with F and enhanced the formation of apatite. Thus, the primary setting occurred faster, and apatite covered the cement surface. However, this latter event prevented the crystallization of the internal layers and decreased the final setting.

The assessment of the release of calcium ions from CEM cement showed that calcium ion release significantly increased from day one to 14 in all the CEM cement groups, and the incorporation of salts had no adverse effects on this process. In all the groups, pH increased over time (7.6 on day one to 9.3 on day seven, then remaining constant up to day 14). The absence of any increase in pH from day seven to 14 was probably due to the presence of calcium-based compounds, which decreases the ionization of calcium hydroxide and results in the production of lower amounts of calcium and hydroxyl ions.^32^

Calcium sulfate, calcium phosphate, calcium carbonate, and calcium hydroxide form after mixing of CEM cement with water. The formation of these compounds was confirmed by XRD in the present study. Calcium hydroxide breaks down into calcium and hydroxyl ions, which increases the pH and concentration of calcium ion. Moreover, calcium and phosphorus ions are released from the primary cement, resulting in an environment rich in calcium, hydroxyl, and phosphate ions. These ions enhance the formation of hydroxyapatite. SEM micrographs and XRD results confirmed the formation of hydroxyapatite on the surface of the cement. Further studies are required to assess the effect of the incorporation of these salts on other properties of MTA and CEM cement, including their strength and biocompatibility.

## Conclusion

Within the limitations of this study, it was concluded that the incorporation of CaCl_2_ and CaO effectively decreased the setting time of MTA, and the incorporation of Ca(NO_3_)_2_ decreased the setting time of CEM cement.

## Acknowledgments

The authors acknowledge that financial support (0416/61) was provided by the Endodontic Research Center, Research Institute of Dental Sciences, School of Dentistry, Shahid Beheshti University of Medical Sciences, Tehran, Iran. We would like to thank Ali Kamali (Shiraz University) for his comments in this project.

## Authors’ Contributions

FJZ was responsible for the experiment design and performed the experiments in partial fulfillment of requirements for a degree, and wrote the manuscript. MM was responsible for the experiment design and contributed to the discussion. MDcontributed to the discussion. HN conceived the idea, hypothesis, and the experiment design, and contributed to the discussion. All authors have read and approved the final manuscript.

## Funding

Financial support (0416/61) was provided by the Endodontic Research Center, Research Institute of Dental Sciences, School of Dentistry, Shahid Beheshti University of Medical Sciences, Tehran, Iran.

## Competing Interests

The authors declare no competing interests with regards to the authorship and/or publication of this article.

## Ethics Approval

Not applicable.
